# Upgrade to malignancy after excision of MRI-only B3 breast lesions: should the size and histological type of the lesion be considered for therapeutic management?

**DOI:** 10.1186/s13244-025-02177-1

**Published:** 2026-01-12

**Authors:** Javier del Riego, Claudia Estandía, Cecilia Aynes, Adriana Campmany, Fiona Pallarés, Sergi Triginer, Natalia Papaleo, Aida López, Oscar Aparicio, Elsa Dalmau, Lidia Tortajada

**Affiliations:** 1 Women’s Imaging, Department of Radiology, Department of Diagnostic Imaging, UDIAT Centre Diagnòstic, Parc Taulí Hospital Universitari, Institut d’Investigació i Innovació Parc Tauli I3PT, Universitat Autònoma de Barcelona, 1 Parc Tauli, Sabadell, Barcelona, Spain; 2 Women’s Imaging, Grup Duran Diagnòstic per la Imatge, Sabadell, Barcelona, Spain; 3 Department of Radiology, Department of Diagnostic Imaging, UDIAT Centre Diagnòstic, Parc Taulí Hospital Universitari, Institut d’Investigació i Innovació Parc Tauli I3PT, Universitat Autònoma de Barcelona, 1 Parc Tauli, Sabadell, Barcelona, Spain; 4 Department of Pathology, Parc Taulí Hospital Universitari, Institut d’Investigació i Innovació Parc Tauli I3PT, Universitat Autònoma de Barcelona, Barcelona, Spain; 5 Department of Gynecology and Obstetrics, Parc Taulí Hospital Universitari, Institut d’Investigació i Innovació Parc Tauli I3PT, Universitat Autònoma de Barcelona, Barcelona, Spain; 6 Department of Surgery, Parc Taulí Hospital Universitari, Institut d’Investigació i Innovació Parc Tauli I3PT, Universitat Autònoma de Barcelona, Barcelona, Spain; 7 Department of Oncology, Parc Taulí Hospital Universitari, Institut d’Investigació i Innovació Parc Tauli I3PT, Universitat Autònoma de Barcelona, Barcelona, Spain

**Keywords:** High-risk lesions, Breast MRI, MRI-guided biopsy, Breast cancer

## Abstract

**Objectives:**

To determine the rate of malignancy upgrade in MRI-only B3 lesions and to identify clinical, imaging, and histological features that can predict upgrade.

**Materials and methods:**

This retrospective single-center study included MRI-only lesions diagnosed as B3 after MRI-guided vacuum-assisted biopsy and excised between January 2007 and March 2023. We calculated upgrade rates for the entire series and for subgroups based on possible risk factors. To analyze variables considered risk factors for upgrade, we used logistic regression, calculating odds ratios (OR) and their 95% confidence intervals (CI).

**Results:**

Of 592 lesions biopsied, 89 (15.03%) were classified as B3. After excluding 30 lesions because excisional specimen results were unavailable, we analyzed 59 lesions in 51 patients. Biopsy classified 15 (25.4%) lesions as pure atypical ductal hyperplasia (ADH), 27 (45.8%) as pure flat epithelial atypia (FEA), 12 (20.3%) as mixed lesions, and 5 (8.5%) as lobular neoplasia. A total of 7 (11.9%) lesions were upgraded to malignancy (71.4% to ductal carcinoma in situ, 14.3% to invasive ductal carcinoma, and 4.3% to invasive lobular carcinoma). Although histological type was not associated with upgrade to malignancy (*p* = 0.47), 20% of pure ADH and only 3.7% of pure FEA lesions were upgraded. Larger lesion size on MRI was associated with upgrade [6.25% of lesions ≤ 20 mm vs. 36.4% of those > 20 mm, *p* = 0.02; OR 8.57 (95% CI: 1.57‒46.71) *p* = 0.01].

**Conclusion:**

Lesion size may predict upgrade in MRI-only B3 lesions independent of histological type; imaging follow-up may suffice for FEA lesions measuring < 20 mm.

**Critical relevance statement:**

Considering lesion size and histological type could help define the management of MRI-only lesions classified as B3 after MRI-guided vacuum-assisted biopsy.

**Key Points:**

- The management of MRI-only B3 lesions has yet to be established.
- Lesion size is a relevant factor to consider when deciding clinical management in MRI-only B3 lesions.
- Conservative management appears to be safe in selected flat epithelial atypia lesions (< 20 mm).

**Graphical Abstract:**

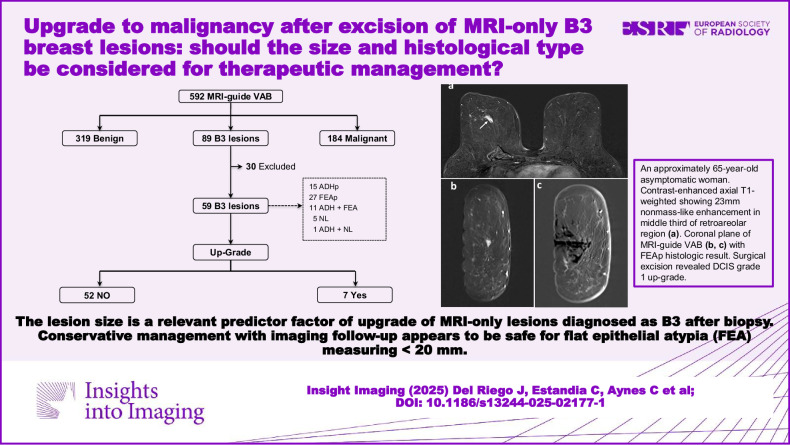

## Introduction

In recent years, the use of contrast-enhanced MRI in the radiological management of breast disease has increased considerably because it is more sensitive than mammography and ultrasound for the detection of invasive cancer, carcinoma in situ, and lesions of uncertain malignant potential [1, 2]. Consequently, the number of MRI-only lesions (i.e., those not visible on other imaging modalities) and MRI-guided vacuum-assisted biopsies to enable the histological study of these lesions has also increased [3].

Between 9 and 16% of MRI-only lesions are classified as lesions of uncertain malignant potential after biopsy [4–7], corresponding to B3 lesions in the National Health Service Breast Screening Programme (NHSBSP) classification system [8]. Some of these lesions share histological characteristics with ductal carcinoma in situ (DCIS) and invasive ductal carcinoma, being considered nonobligatory precursors of malignancy and/or biological indicators of increased risk of developing breast cancer [9, 10].

The management of B3 lesions is a major challenge that requires balancing the risks of overdiagnosis and overtreatment against those of underdiagnosis and undertreatment. The optimal balance has yet to be determined. Although most B3 lesions are managed with vacuum-assisted excision or surgical excision, imaging surveillance has become an option in selected cases where the rate of upgrade is low and diagnostic delay does not affect the patient’s prognosis [11–13].

To date, imaging findings alone have been unable to predict upgrade to malignancy. The few studies that have explored the relationship between MRI findings and upgrade rates have yielded inconclusive results [1, 3, 14, 15]. Because the upgrade rate is higher for MRI-only B3 lesions than in those detected using conventional techniques, current guidelines recommend excision rather than imaging follow-up as the treatment of choice; however, these recommendations are based on small series with scant statistical power [3, 14, 16–18].

This study aims to determine the rate of malignancy upgrade in MRI-only lesions histologically diagnosed as B3 after MRI-guided vacuum-assisted biopsy and to identify clinical, imaging, and histological characteristics that can predict malignancy upgrade.

## Materials and methods

### Study design and population

This retrospective observational study analyzed consecutive cases of MRI-only lesions diagnosed as B3 after MRI-guided vacuum-assisted biopsy at a single center between January 2007 and March 2023. Our hospital’s ethics committee (Code Number: 2023/5072) approved the study, waiving the need for informed consent due to the retrospective nature of the study.

We included MRI-only lesions (undetected on mammography and ultrasound, including on second-look ultrasound when done), histologically classified as B3 after MRI-guided vacuum-assisted biopsy that were analyzed histologically after surgical or percutaneous excision. We excluded lesions treated conservatively and those in patients for whom complete clinical information was unavailable.

### MRI acquisition and image analysis

Images were acquired on 1.5-T MRI scanners with 8- or 16-channel surface coils (Symphony Maestro Class or Magnetom Aera; Siemens Medical Systems). We acquired coronal or axial T2-weighted turbo spin-echo images and dynamic 3D coronal or axial T1-weighted FLASH images of both breasts before and five times after intravenous injection of contrast material (0.16 mmol/kg body weight) with the following parameters: matrix, 512 × 512; FOV entire breast; and slice thickness, 3 mm with isotropic voxels. To evaluate dynamic postcontrast images, we obtained subtracted sequences and multiplanar reconstructions. Although we acquired diffusion-weighted images (DWI) (with b-values 0 and 800) and calculated apparent diffusion coefficients, the information from these images was not used for this study. The most common indications for MRI were local staging, screening high‒risk women, nipple discharge, and breast cancer surveillance.

One of seven radiologists (with 8–22 years of experience in breast radiology, including J.D. and L.T.) interpreted MRI studies, considering patients’ clinical history and findings on other breast imaging studies, including mammograms and ultrasound, when available. To grade lesions’ degree of suspicion, we used the Breast Imaging Reporting and Data System (BI-RADS®) [19]. Lesions classified as BI-RADS 4 or 5 were biopsied percutaneously. Our center’s standardized protocol calls for ultrasound-guided core biopsy when the interpreting radiologist thinks the lesion will likely be visible on second-look ultrasound and for MRI-guided vacuum-assisted biopsy with 9 G needles (ATEC; Suros Surgical Systems, Hologic Inc.) if not. The protocol specifies that radiologists should aim to obtain ≥ 12 specimens in each biopsy procedure, place biopsy site markers, and confirm markers by mammography.

### Pathology techniques

Dedicated breast pathologists with 10 to 25 years’ experience (including N.P.) analyzed specimens, classifying them into five categories according to the NHSBSP system [20], where the following lesion types are classified as having uncertain malignant potential (B3): atypical ductal hyperplasia (ADH); flat epithelial atypia (FEA); lobular neoplasia, which includes lobular carcinoma in situ and atypical lobular hyperplasia; papillary lesions; radial scars; and other miscellaneous entities such as fibroepithelial lesion, mucocele-like lesions, and apocrine adenosis.

Specimens were immediately fixed in buffered formalin, embedded in paraffin, processed with ≥ 3 section-levels, and stained with hematoxylin-eosin following standardized protocols. Cases with malignancy upgrade were classified as DCIS when neoplastic epithelial cells were confined to the ductolobular system and did not extend beyond the basement membrane [21] or as invasive carcinomas according to the World Health Organization classification [21]. Based on immunohistochemistry findings, tumors were classified into the following phenotypes [22]: Luminal A: ER+/−, PR > 20%/−, Ki67 < 20%; Luminal B: ER+/−, PR+ < 20%/−, Ki67 ≥ 20% or ER+; PR+; Her2+; Her2-enriched: ER-, PR-, Her2+; TNBC: ER-, PR-; or Her2-.

### Treatment management

According to our center’s protocol, our multidisciplinary breast disease committee indicated surgical intervention: mastectomy, surgical excision, or percutaneous excision with a breast-lesion excision system (Intact®-BLES; Intact Medical Corporation). Lesions upgraded to malignancy after percutaneous excision were surgically excised to ensure adequate margins.

### Statistical analysis

We analyzed the following variables: patient’s age; presence, histological type, and location of synchronous breast lesions; indication for MRI; type of MRI finding; lesion size (0‒10 mm, 11‒20 mm, > 20 mm); number of biopsy specimens (≤ 12 or > 12); histological type of B3 lesion; and treatment.

The findings on the histological analysis of the specimen from surgical or percutaneous excision were considered the gold standard.

We defined malignancy upgrade as the histological classification of excision specimens as carcinoma (in situ or invasive) after an earlier diagnosis of B3 in the MRI-guided vacuum-assisted biopsy specimen. We calculated the upgrade rate for the entire series and for each type of B3 lesion.

A univariate logistic regression analysis was performed including probable risk factors for malignancy upgrade: age (≤ 50 years or > 50 years), presence of ≥ 1 synchronous lesion, histological type of synchronous lesions, pattern of contrast uptake, lesion size (≤ 20 mm or > 20 mm), number of biopsy specimens, and B3 histological type. We calculated odds ratios (OR) with their 95% confidence intervals. Subsequently, we constructed multivariable logistic regression models including only variables significant at *p* < 0.05.

We report categorical variables as frequencies and percentages and continuous variables as means and standard deviations. To compare lesions that were upgraded versus those that were not, we used chi-square tests for categorical variables and Student’s *t*-tests for continuous variables. Significance was set at *p* < 0.05.

To collect and manage data for this study, we used REDCap® electronic data capture tools hosted at our institution. All statistical analyses were performed using R Statistical Software (R version 4.4.0 and RStudio 2023.09.0 Build 463).

## Results

**Fig. 1:**
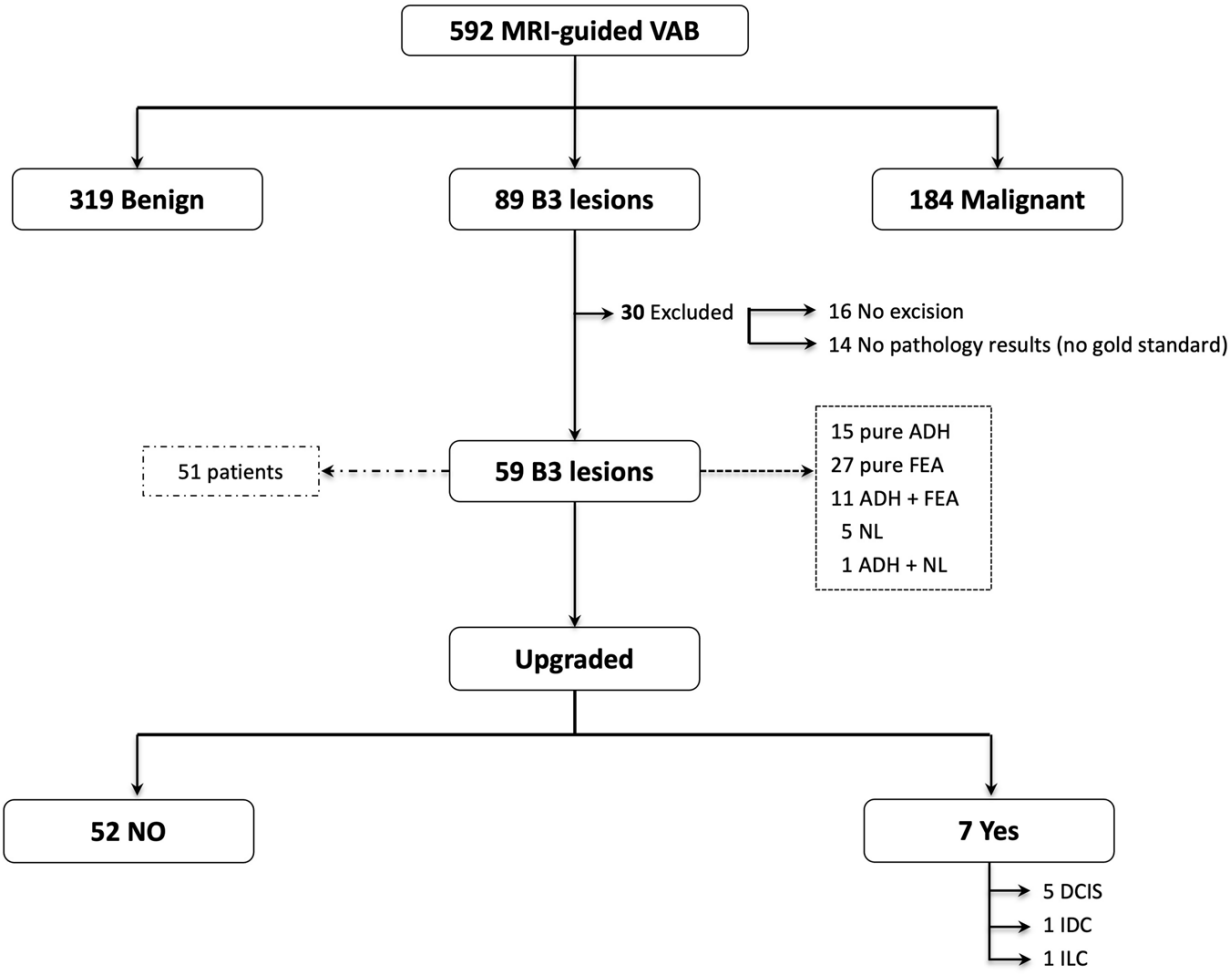
Flowchart showing the inclusion process.VAB, vacuum-assisted biopsy; ADH, atypical ductal hyperplasia; FEA, flat epithelial atypia; LN, lobular neoplasia; DCIS, ductal carcinoma in situ; IDC, invasive ductal carcinoma; ILC, invasive lobular carcinoma

The flowchart in Fig. 1 details the inclusion process. Between January 2007 and March 2023, a total of 592 MRI-guided vacuum-assisted biopsies were performed in 492 patients. Histological study classified 319/592 (53.8%) biopsies as benign, 184/592 (31.1%) as malignant (invasive carcinoma or DCIS), and 89/592 (15.03%) as B3. A total of 30/89 (33.7%) lesions were excluded because gold-standard findings were unavailable; thus, we analyzed 59 B3 lesions in 51 patients (4 patients had ≥ 2 lesions). Of these, 15/59 (25.4%) were classified as pure ADH, 27/59 (45.8%) as pure FEA, 12/59 (20.3%) as mixed lesions (ADH + FEA or ADH+lobular neoplasia), and 5/59 (8.5%) as lobular neoplasia. Table 1 compares demographic, clinical, diagnostic, and treatment variables between B3 lesions upgraded to malignancy and those that were not upgraded.

**Table 1:** Demographics, tumor characteristics, diagnosis, MRI and treatment in the population

	Not upgraded (*n* = 52)	Upgraded (*n* = 7)	*p*-value
Age in years, mean (SD; range)	53 (10.87; 34‒77)	61 (8.52; 48‒73)	
≤ 50 years, *n* (%)	24 (46.1)	1 (14.3)	0.232
> 50 years, *n* (%)	28 (53.9)	6 (85.7)	
Synchronous breast lesion (SBL) detected on MRI, *n* (%)			
Yes	42 (80.8)	6 (85.7)	1
No	20 (19.2)	1 (14.3)	
Histologic result of SBL, *n* (%)			0.11
B3 lesions	10 (19.2)	1 (14.3)	
DCIS	5 (9.7)	2 (28.6)	
LCIS	0 (0.0)	1 (14.3)	
IDC	23 (44.2)	2 (28.6)	
ILC	1 (1.9)	0 (0.0)	
No SBL	13 (25)	1 (14.3)	
Synchronous breast cancer, *n* (%)			0.704
Yes	29 (55.8)	5 (71.4)	
No	23 (44.2)	2 (28.6)	
Location of SBL, *n* (%)			0.928
Ipsilateral	21 (40.4)	3 (42.8)	
Contralateral	20 (38.5)	3 (42.8)	
Bilateral	1 (1.9)	0 (0.0)	
Missing	10 (19.2)	1 (14.3)	
Indications for breast MRI, *n* (%)			0.604
Local staging	40 (76.9)	6 (85.7)	
Screening high‒risk women	4 (7.7)	0 (0.0)	
Nipple discharge	1 (1.9)	0 (0.0)	
Breast cancer surveillance	5 (9.6)	0 (0.0)	
Others	2 (3.8)	1 (14.3)	
Breast MRI findings, *n* (%)			0.519
Focus	8 (15.4)	0 (0.0)	
Masslike enhancement	21 (40.4)	3 (42.8)	
Non-masslike enhancement	23 (44.2)	4 (57.2)	
Lesion size on MRI, mean (SD; min‒max)	15.92 (8.2; 4‒104)	23.29 (15.65; 7‒45)	0.012
≤ 10 mm	27 (52)	3 (42.8)	
11–20 mm	18 (34.6)	0 (0.0)	
> 20 mm	7 (13.4)	4 (57.2)	
Number of biopsy specimens, *n* (%); mean (min-max)	13.62 (9–20)	14 (12–18)	1
≤ 12	21 (40.4)	3 (42.8)	
> 12	22 (42.3)	2 (28.6)	
Missing	9 (17.3)	2 (28.6)	
B3 lesion histologic type, *n* (%)			0.472
Pure FEA	26 (50)	1 (14.3)	
Pure ADH	12 (23.1)	3 (42.8)	
ADH + FEA	9 (17.3)	2 (28.6)	
ADH + LN	1 (1.9)	0 (0.0)	
LN (LCIS included)	4 (7.7)	1 (14.3)	
Treatment, *n* (%)			0.331
Percutaneous excision	1 (1.9)	0 (0.0)	
Surgical excision	46 (88.5)	5 (71.4)	
Mastectomy	5 (9.6)	2 (28.6)	

*SD* standard deviation, *SBL* synchronous breast lesion, *ADH* atypical ductal hyperplasia, *FEA* flat epithelial atypia, *LN* lobular neoplasia (includes lobular carcinoma in situ (LCIS) and atypical lobular hyperplasia)

A total of 7/59 (11.9%) of the B3 lesions were upgraded to malignancy (5/7 (71.4%) DCIS, 1/7 (14.3%) invasive ductal carcinoma, and 1/7 (14.3%) invasive lobular carcinoma); all upgraded lesions were low-grade tumors with Luminal A phenotype. By histological subtype, the upgrade rate was 20% in pure ADH (3/15) and lobular neoplasia (1/5), 18.2% in mixed (FEA + ADH) lesions (2/11), and only 3.7% in pure FEA lesions (1/27), although these differences did not reach statistical significance (*p* = 0.47). Likewise, the difference in the upgrade rates between ADH and non-ADH lesions was not significant (18.5% (5/27) vs. 6.2% (2/32), respectively, *p* = 0.29).

Of the 7 upgraded lesions, 6/7 (85.7%) were synchronous lesions (including B3 lesions or cancers), and 5/7 (71.4%) were synchronous cancers (in situ or invasive carcinoma) (*p* = 0.70).

Lesion size on MRI was the only variable that differed significantly between lesions that were upgraded and those that were not: 3/48 (6.2%) of lesions measuring ≤ 20 mm were upgraded vs. 4/11 (36.4%) of those measuring > 20 mm lesions (*p* = 0.02). In the entire series, 4/7 (57.2%) of the upgraded lesions measured > 20 mm. According to the type of B3 lesions, the upgrade rates for lesions measuring > 20 mm were 20% (1/5) for pure FEA and 33.3% (2/6) for pure or mixed ADH. In the logistic regression analysis, lesion size > 20 mm was a predictive factor for malignancy upgrade regardless of the histological subtype of B3 lesion [OR 8.57 (95% CI: 1.57‒46.71) *p* = 0.01]. Table 2 details the other variables included in the analysis. It was not possible to perform a multivariate analysis because the only variable with *p* < 0.05 was lesion size.

**Table 2:** Variables included in the univariate logistic regression analysis

	Univariate analysis		
	OR	(95% CI)	*p*-value
Age	5.14	(0.58–45.75)	0.142
Synchronous breast lesion	1.43	(0.15–13.23)	0.753
Synchronous breast cancer	1.98	(0.35–11.17)	0.438
Lesion size on MRI	**8.57**	**(1.57–46.71)**	**0.013**
Number of biopsy specimens	0.64	(0.1–4.2)	0.639
B3 lesion histologic type	0.29	(0.05–1.65)	0.165

*OR* odds ratio, *CI* confidence interval

Statistically significant values for the lesion size variable are indicated in bold

## Discussion

MRI-only lesions classified as B3 at histology after biopsy have a higher upgrade rate than B3 lesions diagnosed by conventional breast imaging techniques (mammography or ultrasound), and they are also the most common reason for false-positive results on breast MRI [18, 23]. Liberman et al [18] attribute these findings to the increased risk of cancer among women referred for breast MRI. In our series, B3 lesions accounted for 15% of the lesions biopsied after breast MRI. These results are in line with those of other studies, where the proportion of B3 lesions ranged from 12.7 to 23.3% [1, 3, 4, 14, 16, 24]. In our series, 11.9% of B3 lesions were upgraded (71.4% to low-grade DCIS and 28.6% to invasive carcinoma); this rate is somewhat lower than in other series (range 50‒14%) [1, 3, 4, 14, 16, 18], most likely due to the high proportion of pure FEA lesions in our series. Table 3 summarizes the upgrade rates for B3 lesions in published series.

**Table 3:** Published studies with MRI-guided vacuum-assisted biopsy and B3 lesions

First author [ref.]	MRI-VABB	B3 lesions included (%)	Upgrade (%)	Pathology results for upgraded lesions
Strigel [3]	482*	39 (8.1)	12 (30.8)	7 DCIS; 3 IDC; 1 ILC
Crystal [14]	161	26 (16.1)	13 (50)	11 DCIS; 1 IDC; 1 mixed (IDC – ILC)
Heller [4]	1145	147 (12.8)	30 (20.4)	19 DCIS; 8 IDC; 2 ILC
Weinfurtner [1]	257	29 (11.3)	4 (13.8)	3 DCIS; 1 IDC
Beaulieu-Jones [16]	399	37 (9.3)	9 (24.3)	1 DCIS; 1 mixed (DCIS – IDC); 7 invasive
Okamoto [30]	598	84 (14)	19 (22.6)	12 DCIS; 2 IDC; 1 mixed (IDC – ILC); 4 ILC
Michels [24]	810	159 (19.6)	13 (8.2)	8 DCIS; 3 IDC; 2 ILC
Cha [29]	306	72 (23.5)	8 (11.1)	4 DCIS; 2 IDC; 2 ILC
Current study	592	59 (10)	7 (11.8)	5 DCIS; 1 IDC; 1 ILC

*MRI-VABB* vacuum-assisted breast biopsy, *DCIS* ductal carcinoma in situ, *IDC* invasive ductal carcinoma, *ILC* invasive lobular carcinoma

* Includes biopsies performed using different techniques

Analyzing a series of 151 B3 lesions, Heller et al [4] found an upgrade rate of 19.9%. Interestingly, 70% of the upgraded lesions were ipsilateral synchronous breast cancers (*p* < 0.001). In our series, 85.7% of the upgraded lesions were synchronous lesions (B3 or carcinoma) and 71.4% of these were synchronous cancers (DCIS or invasive carcinoma; *p* = 0.70); although these proportions did not reach statistical significance, we consider that the lack of statistical significance is likely related to the small size of our sample, so concomitant cancer might very well be an important predictive factor for upgrade.

Moreover, like other authors [1, 3, 14, 16, 18, 24], we found no significant associations between upgrade and age, histology of the synchronous lesion, location of the synchronous lesion, indication for MRI, or MRI findings.

The most common histological types of B3 lesions are FEA and ADH [25]; in our series, 45.8% of all B3 lesions were pure FEA, 25.4% were pure ADH, and 18.6% were mixed ADH + FEA. ADH is the B3 lesion with the greatest risk of upgrade, although the proportion of lesions of different histological types that were upgraded varies among series. In a meta-analysis of 93 studies including a total of 6458 lesions diagnosed as ADH after percutaneous biopsy, Schiaffino et al [26] found the overall upgrade rate was 29% [95% CI: 26‒32%] for surgically excised lesions and 5% [95% CI: 4‒8%] for lesions managed with follow-up; the upgrade rate for surgically excised lesions initially diagnosed as ADH after MRI-guided biopsy (used in 9 studies) was 32% (95% CI: 22‒43%). Given the evidence, the main guidelines recommend excision as the treatment of choice for lesions diagnosed as pure or mixed ADH [11–13]. Michaels et al [24] found that 22.5% of the 40 B3 lesions diagnosed after MRI-guided biopsy in their series were upgraded; pooling their data with those from 10 other studies to include a total of 88 ADH diagnosed after MRI-guided biopsies, they found a total upgrade rate of 24.6% (range, 15‒53.3%). In our series, the upgrade rate was 20% for pure ADH and 16.7% in mixed lesions with an ADH component. The difference in the upgrade rates for lesions with an ADH component versus those with no ADH component (18.5% vs. 6.2%, respectively, *p* = 0.29) did not reach statistical significance, probably due to the low number of cases.

Unlike in other studies, our series had a high proportion of FEA; to our knowledge, our series contained the highest proportion of pure FEA (45.8%) among series diagnosed with MRI-guided vacuum-assisted biopsy published to date. This finding is undoubtedly influenced by the wide variability in the histological diagnosis of FEA among different pathologists [27]. Of the 27 pure FEA lesions in our series, only 1 (3.7%) was upgraded; this finding is in line with the results published for other studies. Michaels et al [24] found that only 1 of the 3 FEA lesions in their study was upgraded; pooling their data with those from 4 other studies to include a total of 31 FEA diagnosed after MRI-guided biopsies yielded an upgrade rate of 3.2%. Although BI-RADS advises surgical excision for all lesions with a risk of malignancy > 2% [19], various consensus statements and international guidelines accept imaging follow-up for lesions of uncertain malignant potential with an upper limit underestimation rate of 5% for invasive carcinoma and 10% for DCIS [11–13, 28].

The most noteworthy finding in our study is the association between lesion size on MRI and the risk of upgrade. The upgrade rate was significantly higher in lesions measuring > 20 mm than in those measuring ≤ 20 mm [36.4% vs. 6.2%, *p* = 0.01]. In our series, 4 (57.2%) of the 7 lesions that were upgraded measured > 20 mm. To our knowledge, ours is the first study to report a significant association between lesion size and the risk of upgrade in MRI-only B3 lesions. Although other studies showed trends toward greater risk of upgrade among larger lesions, these associations did not reach statistical significance at *p* < 0.05 [1, 3, 4, 14, 16, 18, 24, 29, 30]. In a series of 72 B3 MRI-only lesions in which 8 (11.1%) lesions were upgraded, Cha et al [29] reported a trend (*p* = 0.089) toward larger size among upgraded lesions (mean 21.3 mm vs. 9.7 mm in lesions not upgraded); notably, this series did not include any cases of FEA.

Importantly, our logistic regression analysis found that MRI lesion size > 20 mm significantly increased the risk of upgrade [OR 8.57 (95% CI: 1.57‒46.71) *p* = 0.01]. This result suggests a strong association between lesion size and the risk of upgrade, despite the wide confidence interval due to the low number of upgraded lesions in our study. Studies including larger series are necessary to achieve greater statistical power.

**Fig. 2:**
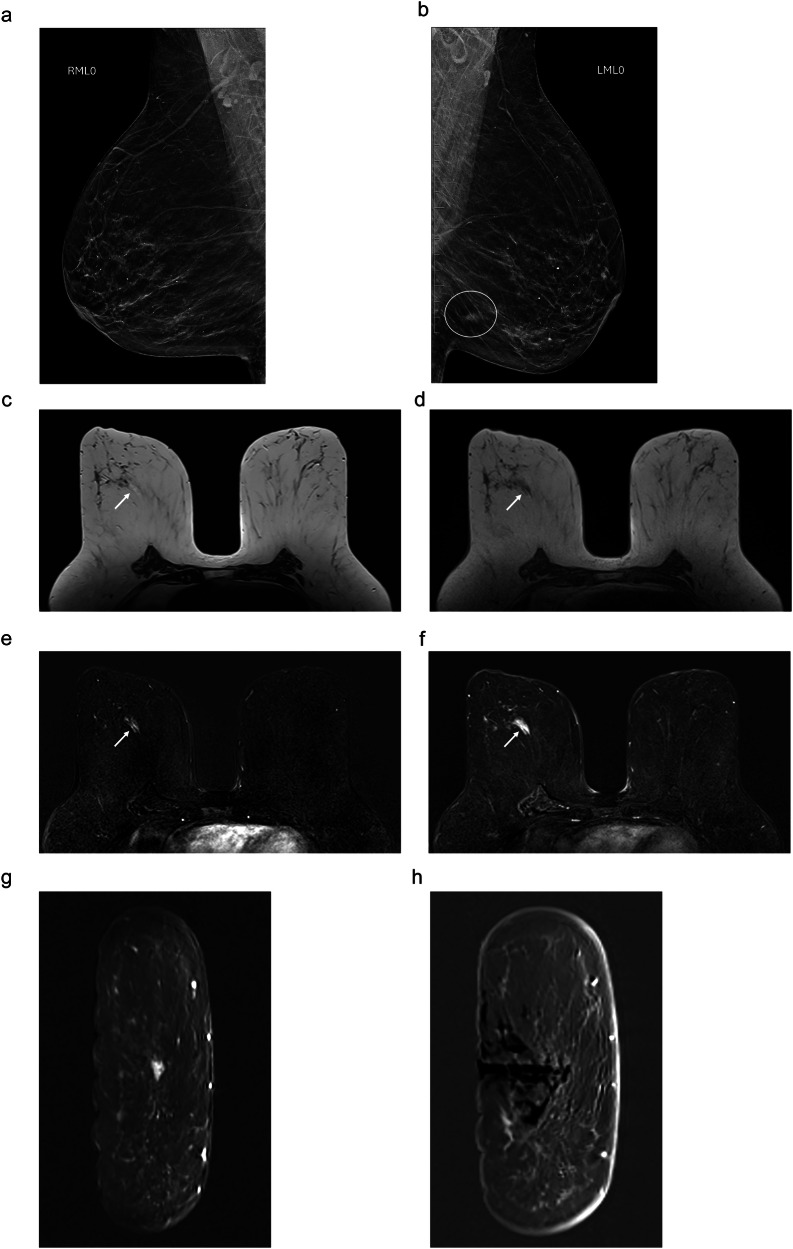
MRI-only lesion diagnosed as pure FEA after biopsy and upgraded to malignancy after excision in an asymptomatic 64-year-old woman.Screening mammogram: **a** right mediolateral oblique projection shows no suspicious findings; **b** left mediolateral oblique projection shows an irregular mass (circle) in the posterior third of the lower quadrants. Percutaneous biopsy diagnosed a luminal A grade 2 invasive ductal carcinoma. Staging MRI: **c** axial T2-weighted; **d** unenhanced axial T1-weighted; **e**, **f** subtracted contrast-enhanced axial T1-weighted image obtained 2 min and 4 min after contrast injection shows a 23 mm area of progressive non-masslike enhancement in the middle third of the retroareolar region. **g**, **h** Coronal MRI used for vacuum-assisted biopsy. The biopsy specimen was diagnosed as pure FEA; histological analysis of the excised lesion upgraded the diagnosis to grade 1 DCIS

Radiologists should be aware of the importance of lesion size and consider it together with other variables when determining the therapeutic management of MRI-only B3 lesions. Although vacuum-assisted biopsy with 9 G needles enables more material to be obtained than in core biopsies, it does not enable complete excision in lesions measuring > 20 mm, thus introducing a risk of underdiagnosis. In line with guidelines and consensus statements about the management of B3 lesions diagnosed with techniques other than MRI-guided vacuum-assisted biopsy [11, 12], we believe that localized surgical excision should also be the treatment of choice for lesions > 20 mm diagnosed with this technique to guarantee representative sampling and rule out malignancy. It is worth pointing out that the only pure FEA that was upgraded (to low-grade DCIS) after surgical excision was a lesion with non-masslike enhancement that measured 23 mm in maximum diameter (Fig. 2). For this reason, we consider that MRI (always together with conventional imaging modalities) could be indicated for the follow-up of MRI-only FEA lesions measuring < 20 mm.

Our study adds to the growing body of evidence about MRI-only B3 lesions. Nevertheless, various limitations of our study must be taken into account. In addition to the limitations common to all retrospective studies, MRI studies were not retrospectively reviewed. Thus, our data came from image interpretation during routine clinical practice in which radiologists were not blinded to clinical information and did not obtain a standardized number of specimens in biopsy procedures. Moreover, the small sample, which did not even contain all types of B3 lesions, made it difficult to obtain statistically significant results, especially in the analysis of histological types of B3 lesions, where we observed a marked trend toward upgrade in ADH lesions. Additionally, not including risk factors such as prior history of breast cancer or genetic syndromes may potentially have introduced a confounding bias. Moreover, in analyzing MRI features, we did not consider DWI; the specificity of DWI and ADC for differentiating among breast lesions is greater than that of dynamic contrast-enhanced MRI alone, and including findings derived from DWI might improve predictions regarding the risk of upgrade. Cheeney et al [31] found lower ADC values in B3 lesions that were upgraded after surgical excision. Although DWI promises to play an increasingly important role in breast imaging, the significance of different DWI findings in B3 lesions remains to be determined. Finally, the data were collected at a single center over a relatively long period of time, so caution is warranted in extrapolating our findings to other contexts.

## Conclusion

In MRI-only lesions diagnosed as B3 after biopsy, lesion size seems to be a relevant predictive factor for upgrade, and considering lesion size and type could help guide management decisions. Likewise, the presence of synchronous cancer might very well also be a predictive factor for upgrade. Our results suggest surgical excision is the treatment of choice for lesions measuring > 20 mm, regardless of their histological type, as well as for all ADH lesions. Conservative management with imaging follow-up appears to be safe for FEA measuring < 20 mm. Studies with larger cohorts and meta-analyses are needed to enable management based on stronger evidence.

## Data Availability

The datasets generated and analyzed during the current study are not publicly available but are available from the corresponding author on reasonable request.

## Abbreviations

ADH: Atypical ductal hyperplasia
DCIS: Ductal carcinoma in situ
FEA: Flat epithelial atypia
